# Genome-wide identification and expression analysis of YTH domain-containing RNA-binding protein family in common wheat

**DOI:** 10.1186/s12870-020-02505-1

**Published:** 2020-06-23

**Authors:** Jing Sun, Xiao Min Bie, Ning Wang, Xian Sheng Zhang, Xin-Qi Gao

**Affiliations:** National Key Laboratory of Crop Biology, College of Life Sciences, Shandong Agricultural University, Tai’an, 271018 China

**Keywords:** Wheat, YTH domain-containing RNA-binding protein, Expression profiling, Development, Abiotic stress

## Abstract

**Background:**

N6-Methyladenosine (m6A) is the most widespread RNA modification that plays roles in the regulation of genes and genome stability. YT521-B homology (YTH) domain-containing RNA-binding proteins are important RNA binding proteins that affect the fate of m6A-containing RNA by binding m6A. Little is known about the *YTH* genes in common wheat (*Triticum aestivum* L.), one of the most important crops for humans.

**Results:**

A total of 39 *TaYTH* genes were identified in common wheat, which are comprised of 13 homologous triads, and could be mapped in 18 out of the 21 chromosomes. A phylogenetic analysis revealed that the TaYTHs could be divided into two groups: YTHDF (TaDF) and YTHDC (TaDC). The TaYTHs in the same group share similar motif distributions and domain organizations, which indicates functional similarity between the closely related TaYTHs. The TaDF proteins share only one domain, which is the YTH domain. In contrast, the TaDCs possess three C3H1-type zinc finger repeats at their N-termini in addition to their central YTH domain. In TaDFs, the predicated aromatic cage pocket that binds the methylysine residue of m6A is composed of tryptophan, tryptophan, and tryptophan (WWW). In contrast, the aromatic cage pocket in the TaDCs is composed of tryptophan, tryptophan, and tyrosine (WWY). In addition to the general aspartic acid or asparagine residue used to form a hydrogen bond with N^1^ of m6A, histidine might be utilized in some TaDFb proteins. An analysis of the expression using both online RNA-Seq data and quantitative real-time PCR verification revealed that the *TaDFa* and *TaDFb* genes are highly expressed in various tissues/organs compared with that of *TaDFcs* and *TaDCs*. In addition, the expression of the *TaYTH* genes is changed in response to various abiotic stresses.

**Conclusions:**

In this study, we identified 39 *TaYTH* genes from common wheat. The phylogenetic structure, chromosome distribution, and patterns of expression of these genes and their protein structures were analyzed. Our results provide a foundation for the functional analysis of *TaYTH*s in the future.

## Background

N6-Methyladenosine (m6A) is the most comment posttranscriptional modification in eukaryotic RNAs, which is crucial for gene regulation and the maintenance of genome stability [1]. m6A is a reversible and dynamic modification that is catalyzed by a methyltransferase complex (Writers), such as METTL3, METTL14, WTAP, and RBM15/RBM15B, and removed by demethylases (Erasers), including FTO and ALKBH5. The modification of m6A plays important roles in diverse physiological processes. For example, MTA, MTB, FIP37, and VIR, the homologs of methyltransferase complex subunits in plants, function in the regulation of shoot stem cell fates, root growth, gravitropic responses, and embryo development [2–5]. m6A plays its roles at the molecular level by affecting the formation of secondary RNA structure, during which m6A modification controls the accessibility of binding sites for RNA binding proteins (Readers) [6]. The recognition of m6A by the readers is important for the metabolism, processing, and folding of RNA, and protein translation. Many m6A reader proteins have been identified, and most contain a YT521-B homologous (YTH) domain with an aromatic cage that specifically recognizes the GG(m6A) C sequence [7]. Five YTH domain-containing RNA-binding proteins in humans were identified, and they can be classified into three categories based on their sequences: YTHDC1 (YTH domain-containing protein 1), YTHDC2 (YTH domain-containing protein 2), and YTHDF (YTH domain-containing family protein) family [8]. YTHDF proteins (DF1, DF2, and DF3) are localized in the cytoplasm and contain a C-terminal YTH domain and a large, low-complexity region. It has been shown that YTHDFs bind all the m6A sites in mRNA. YTHDC1 is nuclear-enriched and contains a YTH domain at its central part and is composed of other multiple functional domains. In contrast, YTHDC2 is a nucleocytoplasmic protein that contains a C-terminal YTH domain and a DEAD-box RNA helicase domain. YTHDC1 binds to certain m6A sites in mRNAs and to noncoding RNAs, whereas YTHDC2 primarily binds to noncoding RNAs [9]. Many *YTH* genes have been identified in various plant species, including *Arabidopsis thaliana*, *Oryza sativa*, *Malus domestica*, *Citrus sinensis* and *Cucumis sativus* [10–13]. Thirteen *ECT genes* of *A. thaliana*, homologs of *YTHDFs*, were identified, some of which function in controlling the timing of the first true leaf formation, proper leaf morphology, and trichome branching [14–17]. ECT2 can interact with m6A-containing RNAs in vivo, indicating that YTH domain is an m6A reading domain in *Arabidopsis* [15]. AtCPSF30 is a homolog of human HYTDC, which is a cleavage and polyadenylation specificity factor that functions in oxidative and nitrate signaling [18, 19]. In *A. thaliana*, *M. hupehensis* and *C. sativus*, *YTH* genes are involved in plant responses to pathogens, plant hormones (salicylic acid and abscisic acid), and abiotic stresses (water, temperature, and salinity) [10–13, 20–22]. These studies revealed that YTH domain-containing proteins play important roles in plant development and biotic and abiotic stress responses. Wheat is one of the major cereal crops in the world. To our knowledge, the YTH protein family in common wheat (*Triticum aestivum* L.) has not yet been analyzed in detail. In this study, we identified 39 genes that encode YTH domain-containing proteins in common wheat and analyzed their phylogenetic relationship and expression in different tissues/organs and in response to various stresses.

## Results

### Identification and phylogenetic analysis of the YTH proteins in common wheat

**Fig. 1:**
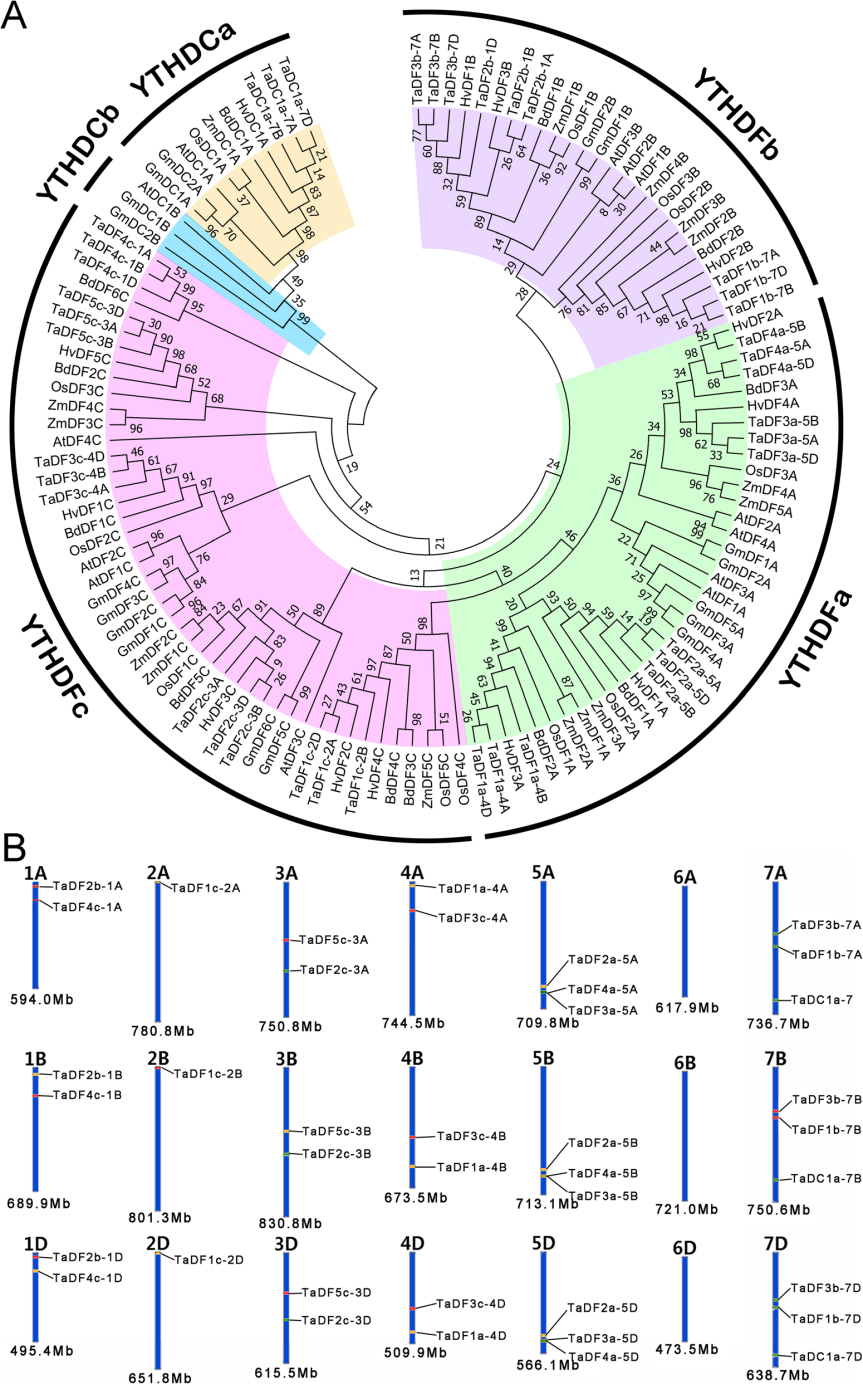
Phylogenetic tree and chromosomal distribution of *TaYTH* genes. **a**. Phylogenetic tree of YTH proteins based on the YTH domains of the YTH proteins from wheat, *O. sativa, A. thaliana, Z. mays*, and *G. max*. **b**. Chromosomal distribution of *TaYTH* genes. All wheat chromosomes are drawn to scale based on their actual lengths

To identify the *TaYTH* genes in common wheat, we used the hidden Markov model profiles of the YTH domain (PF04146) as queries to search the protein databases (IWGSC RefSeq v1.1) of common wheat (https://www.ebi.ac.uk/Tools/hmmer/search/hmmsearch) that was downloaded as a local protein database of bread wheat. Additionally, the conserved YTH domain was also used to BLASTp the YTH proteins encoded by the wheat genome on the website http://plants.ensembl.org/Multi/Tools/Blast [8]. Additionally, we performed BLASTP analysis against the local wheat protein database (E-value < 1 × 10^− 5^) by using the YTH domain-containing protein sequences of *Arabidopsis thaliana* and *Oryza sativa* as queries. After the redundant sequences were removed, a total of 39 putative YTH proteins were identified and used for further analysis (Additional file 1). Their encoding genes are 13 sets of homeoalleles with 13 *YTH* genes in each sub-genome. Additionally, 13, 17, 12, 15, 12, 13 *YTH* genes in *A. thaliana*, *Glycine max*, *O. sativa*, *Zea mays*, *Brachypodium distachyon*, and *Hordeum vulgare*, respectively, were identified using the same strategy in this study and other study [15]. An unrooted phylogenetic tree of 39 *TaYTH* genes was constructed based on the YTH domains of the 39 TaYTHs in concert with 82 YTH proteins from *A. thaliana*, *O. sativa*, *G. max*, *Z. mays*, *B. distachyon*, and *H. vulgare* (Additional file 2) to analyze the evolutionary relationships of the TaYTH proteins (Fig. 1a). The phylogenetic analysis revealed that the TaYTH domain proteins were clustered into two groups: YTHDF (TaDF) and YTHDC (TaDC). The YTHDF family contains three subgroups, YTHDFa, YTHDFb, and YTHDFc, which include 12, 9, and 15 members, respectively. The YTHDC family can be divided into two additional subgroups, YTHDCa and YTHDCb. No YTHDCb proteins were identified in common wheat, which is consistent with the previous hypothesis that there are no group-b YTHDCs in monocotyledons [15]. All of the 39 YTH proteins that had been identified were re-named based on their phylogenetic relationship consistent with the YTH proteins that have been identified in other plant species [15]. The subgenome (A, B or D) on which the genes are located is included in the name, and the number before the subgenome indicates the serial number of chromosome. Chromosomal location information of the *TaYTH* genes was extracted from the GFF3 reference file of the wheat genome, and a chromosomal map was constructed using KnetMiner, an online analytical tool (https://knetminer.rothamsted.ac.uk/Triticum_aestivum/). The map constructed revealed that the *TaYTHs* are located on all chromosomes with the exception of chromosomes 6A, 6B, and 6D. There are three *TaYTH* genes on chromosomes 5A, 5B, 5D, 7A, 7B, and 7D, and two on chromosomes 1A, 1B, 1D, 3A, 3B, 3D, 4A, 4B, and 4D. In contrast, only one *TaYTH* gene is located on each 2A, 2B, and 2D chromosome (Fig. 1b).

### Gene structure and synteny of the *TaYTH* genes

**Fig. 2:**
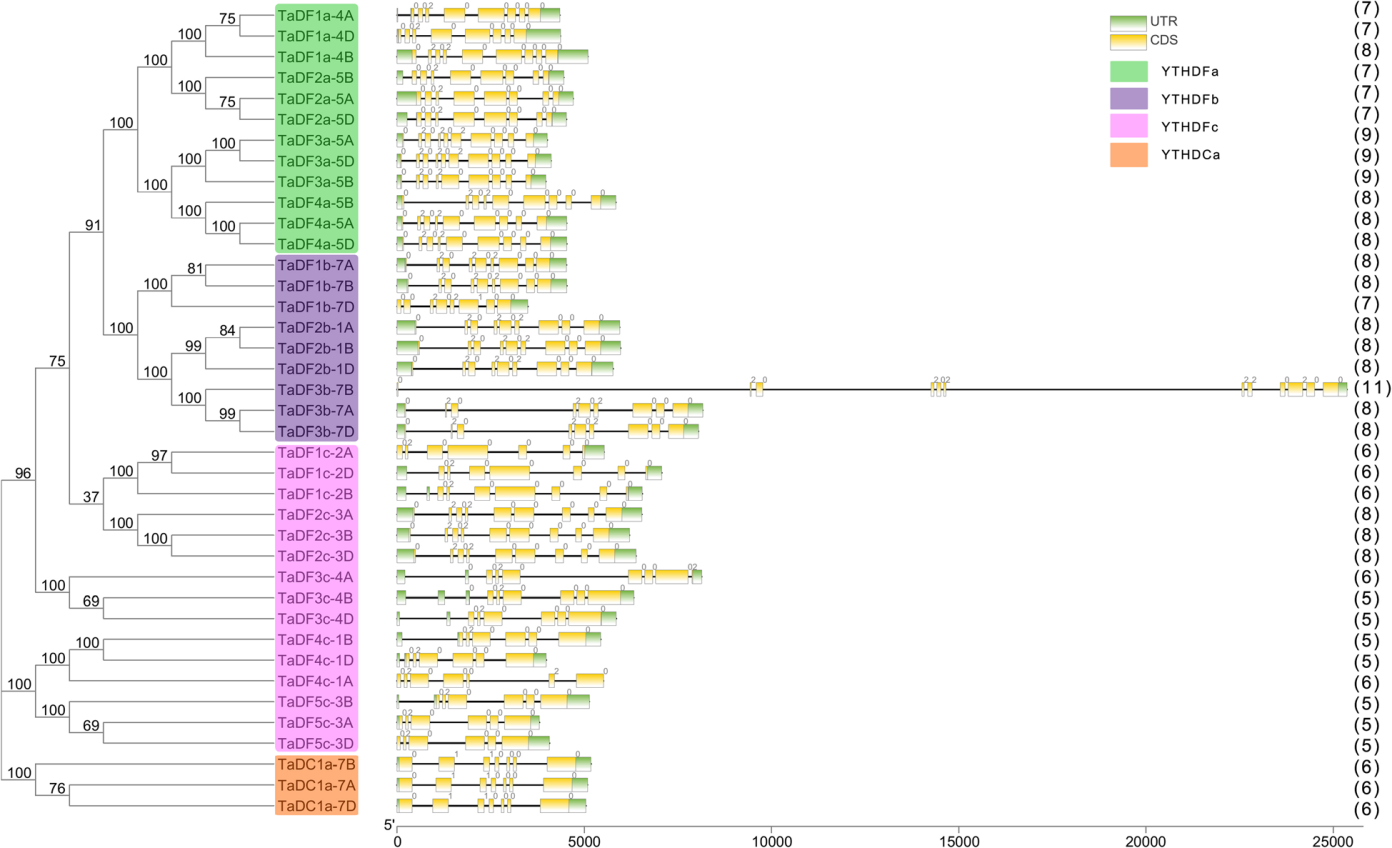
Exon-intron structures and intron phases of *TaYTH* genes. Yellow box, green box, and black line indicate exon, UTR, and intron, respectively. The numbers in brackets are the numbers of intron of TaYTH genes. The phases of intron (0, 1, and 2) are shown on the top of the corresponding introns

The exon-intron structure provides insight on the evolution of gene families and additional evidence to support phylogenetic grouping. We then analyzed the exon-intron structure of the *TaYTH* genes based on their evolutionary classification. All the *TaYTH* genes contain introns, and most of the genes contained from five to eight introns (Fig. 2). In contrast, *TaDF3b-7B* contains the largest number of introns (11), including three long ones (> 4 Kb) that are not found in the *YTH* genes of other plant species [11, 12] (Fig. 2). The patterns of distribution of the *TaYTH* exon/introns were similar among the members within each group in the phylogenetic tree. The proportions of intron phases 1, 2, and 0 are 2.55, 23.3, and 74.2%, respectively, of all the *TaYTH* genes (Fig. 2). There are similar patterns of the intron phases of *TaYTH* genes with those of the *YTH* genes in *A. thaliana, O. sativa* and other species [11], i.e., the largest proportion of introns was in phase 0, with a few in phase 1 (Fig. 2).

**Fig. 3:**
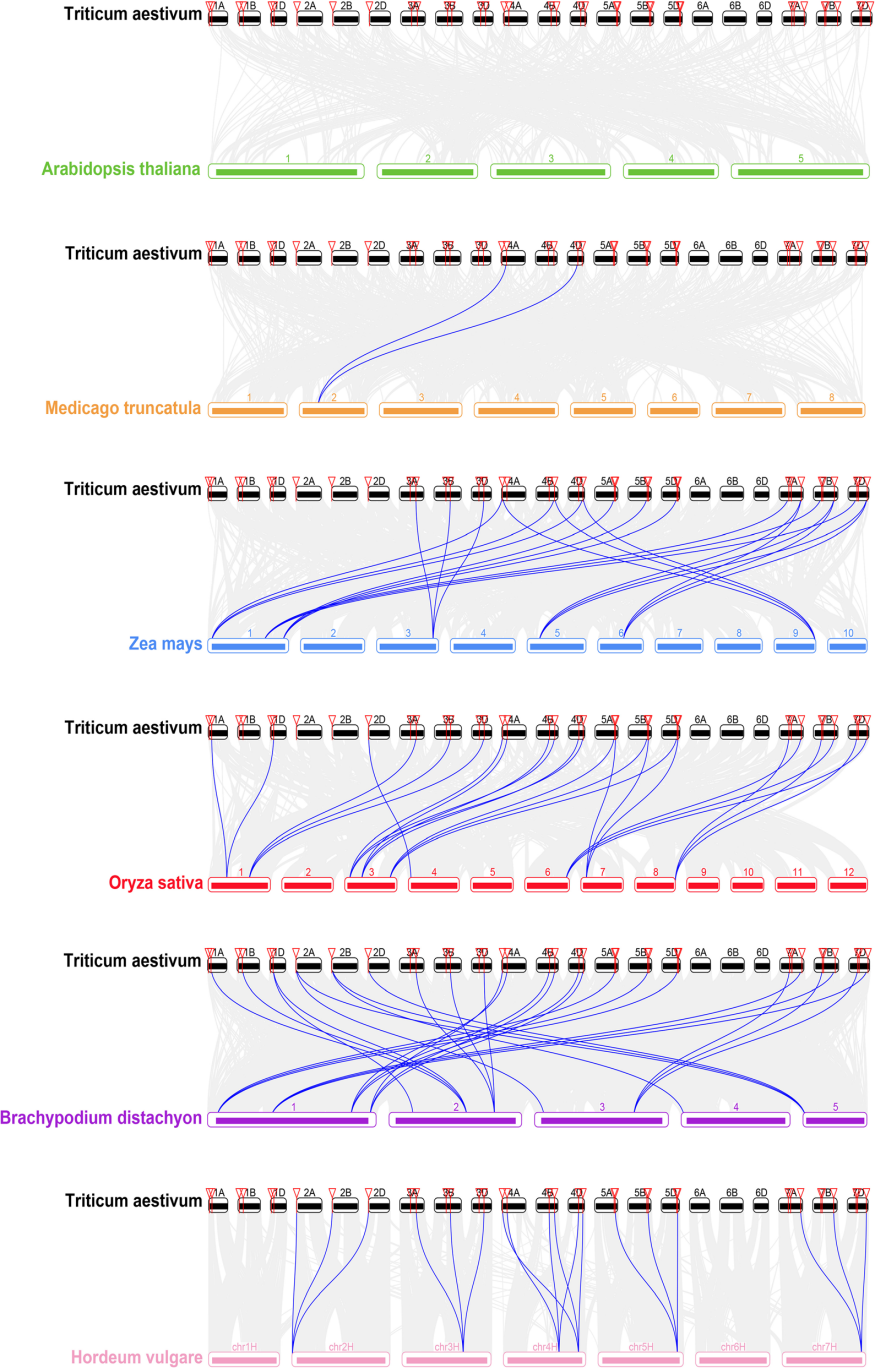
Synteny analysis of *YTH* genes between wheat and other plant species, *A. thaliana*; *O. sativa; Z. mays; M. truncatula* and *B. distachyon*. Gray lines in the background indicate the collinear blocks within the genomes of wheat and other plants, and the blue lines indicate the syntenic *YTH* gene pairs

Genomic replication events often result in the expansion of the gene family during the evolution of angiosperms [23]. Three pairs of genes, *TaDF3a-5A* and *TaDF4a-5A*, *TaDF3a-5B* and *TaDF4a-5B*, and *TaDF3a-5D* and *TaDF4a-5D*, are localized in the region within a 200 kb range in chromosomes 5A, 5B, and 5D, respectively (Fig. 1b, Table 1). In addition, these genes share similar exon-intron structure (Fig. 2). These suggest that these *YTH* genes may be produced by tandem gene replication. The synteny diagram of the *YTH* genes between wheat and other plant species (*A. thaliana*; *O. sativa; Z. mays*; *Medicago truncatula* and *B. distachyon*) revealed that the *YTH* genes of wheat contained the greatest number of homologous gene pairs with those of *B. distachyon*, followed by rice and maize (Fig. 3). No *YTH* homologous gene pairs were identified between wheat and *Arabidopsis*. Only two homologous gene pairs were identified between wheat and *M. truncatula*. In contrast, 18, 21, 21, and 24 *YTH* gene pairs were identified between barley and wheat, rice and wheat, maize and wheat, *B. distachyon* and wheat, respectively, matching the three expected homologous counterparts in wheat (Fig. 3, Additional file 3). Our results reveals there is a similar copy number of *YTH* genes in different species and similar numbers of *YTH* gene pairs among different species and wheat, which suggest that the *YTH* gene family is conservative and withstand strong selection pressure during plant evolution.

**Table 1:** Information of *TaYTH* genes and the predicated proteins in common wheat

Group	Subgroup	Name	Transcript ID	Chromosome location	AA^a^	pI^b^	MW (KDa)^c^	Subcellular localization
YTHDF	DFa	TaDF1a-4A	TraesCS4A02G031600.2	4AS:24753423–24,757,771	729	8.04	78.7	Nucleus
		TaDF1a-4D	TraesCS4D02G273200.4	4DL:442903537–442,907,900	723	8.31	78.1	Nucleus
		TaDF1a-4B	TraesCS4B02G274500.2	4BL:552029023–552,034,120	767	7.58	82.1	Nucleus
		TaDF2a-5B	TraesCS5B02G391500.1	5BL:570890408–570,894,862	662	8.54	71.8	Nucleus
		TaDF2a-5A	TraesCS5A02G386600.2	5AL:584415361–584,420,062	663	8.37	71.8	Nucleus
		TaDF2a-5D	TraesCS5D02G396400.3	5DL:464017988–464,022,514	662	8.54	71.7	Nucleus
		TaDF3a-5A	TraesCS5A02G431200.1	5AL:615184764–615,188,775	589	6.05	64.1	Nucleus
		TaDF3a-5D	TraesCS5D02G439100.1	5DL:492720111–492,724,223	588	6.06	63.9	Nucleus
		TaDF3a-5B	TraesCS5B02G433000.1	5BL:607484742–607,488,713	596	6.77	64.7	Nucleus
		TaDF4a-5B	TraesCS5B02G432900.2	5BL:607473731–607,479,573	648	8.57	70.1	Nucleus
		TaDF4a-5A	TraesCS5A02G431100.1	5AL:615177191–615,181,721	642	8.52	69.5	Nucleus
		TaDF4a-5D	TraesCS5D02G439200.1	5DL:492726393–492,730,926	642	8.52	69.5	Nucleus
	DFb	TaDF1b-7A	TraesCS7A02G291700.1	7A:359048874–359,053,398	590	5.77	65.7	Nucleus
		TaDF1b-7B	TraesCS7B02G182100.2	7BS:282328800–282,333,335	592	5.57	65.5	Nucleus
		TaDF1b-7D	TraesCS7D02G285000.1	7DS:306457509–306,462,059	612	5.55	69.0	Cytoplasm
		TaDF2b-1A	TraesCS1A02G044500.1	1AS:25539196–25,545,141	642	5.40	70.4	Nucleus
		TaDF2b-1B	TraesCS1B02G057800.3	1BS:39847538–39,853,507	642	5.40	70.7	Nucleus
		TaDF2b-1D	TraesCS1D02G045100.1	1DS:24533367–24,539,137	642	5.34	70.5	Nucleus
		TaDF3b-7B	TraesCS7B02G174500.1	7BS:245593610–245,618,979	599	6.05	66.2	Nucleus
		TaDF3b-7A	TraesCS7A02G276800.3	7AS:289606810–289,614,972	625	5.75	69.3	Nucleus
		TaDF3b-7D	TraesCS7D02G276800.4	7DS:265165030–265,173,079	625	5.87	69.3	Nucleus
	DFc	TaDF1c-2A	TraesCS2A02G011400.1	2AS:4215799–4,221,330	711	6.53	80.1	Nucleus
		TaDF1c-2D	TraesCS2D02G012200.1	2DS:5761715–5,768,783	710	6.51	80.0	Nucleus
		TaDF1c-2B	TraesCS2B02G010200.1	2BS:5660528–5,667,076	714	6.91	80.4	Nucleus
		TaDF2c-3A	TraesCS3A02G267700.1	3AL:493127515–493,134,054	691	5.41	75.3	Cytoplasm
		TaDF2c-3B	TraesCS3B02G301100.1	3B:482993891–483,000,099	688	5.49	75.2	Cytoplasm
		TaDF2c-3D	TraesCS3D02G267900.1	3DL:371891101–371,897,485	688	5.69	75.1	Cytoplasm
		TaDF3c-4A	TraesCS4A02G125600.3	4AS:162071718–162,079,853	719	5.74	77.6	Nucleus
		TaDF3c-4B	TraesCS4B02G179100.3	4BL:392107576–392,113,896	714	6.01	77.1	Nucleus
		TaDF3c-4D	TraesCS4D02G180500.5	4DL:314401595–314,407,948	713	5.84	76.9	Nucleus
		TaDF4c-1B	TraesCS1B02G128700.1	1BS:158309306–158,314,745	703	8.86	77.8	Cytoplasm
		TaDF4c-1D	TraesCS1D02G109300.1	IDS:102773595–102,777,581	706	8.75	78.3	Nucleus
		TaDF4c-1A	TraesCS1A02G105700.1	1AS:102164806–102,170,320	702	8.71	77.8	Nucleus
		TaDF5c-3B	TraesCS3B02G232300.2	3B:354942908–354,948,042	687	7.27	75.8	Nucleus
		TaDF5c-3A	TraesCS3A02G199100.2	3AL:323099741–323,104,616	689	6.68	75.9	Nucleus
		TaDF5c-3D	TraesCS3D02G199400.2	3DS:225522840–225,528,001	688	7.02	75.8	Nucleus
YTHDC	DCa	TaDC1a-7B	TraesCS7B02G363200.2	7BL:625715946–625,721,126	653	6.42	71.7	Nucleus
		TaDC1a-7A	TraesCS7A02G461800.1	7AL:657901780–657,906,867	650	6.42	71.3	Nucleus
		TaDC1a-7D	TraesCS7D02G450100.1	7DL:569047266–569,052,310	650	6.46	71.3	Nucleus

^a^*AA* Number of amino acids; ^b^*MW* Molecular weight; ^c^*pI* Isoelectric point

### Protein features of TaYTHs

The lengths of TaYTHs ranged from 588 to 767 amino acid residues. The molecular weight (MW) and isoelectric point (pI) of the TaYTH proteins varied from 63.9 to 82.1 kDa and from 5.40 to 8.54, respectively (Table 1). The subcellular localization of TaYTH proteins was predicted by the online tool PSORT (https://www.psort.org/). TaDF1b-7D, TaDF2c-3A, TaDF2c-3B, TaDF2c-3D, and TaDF4c-1B were predicted to be localized in the cytoplasm, while other TaYTHs were localized in nucleus (Table 1).

**Fig. 4:**
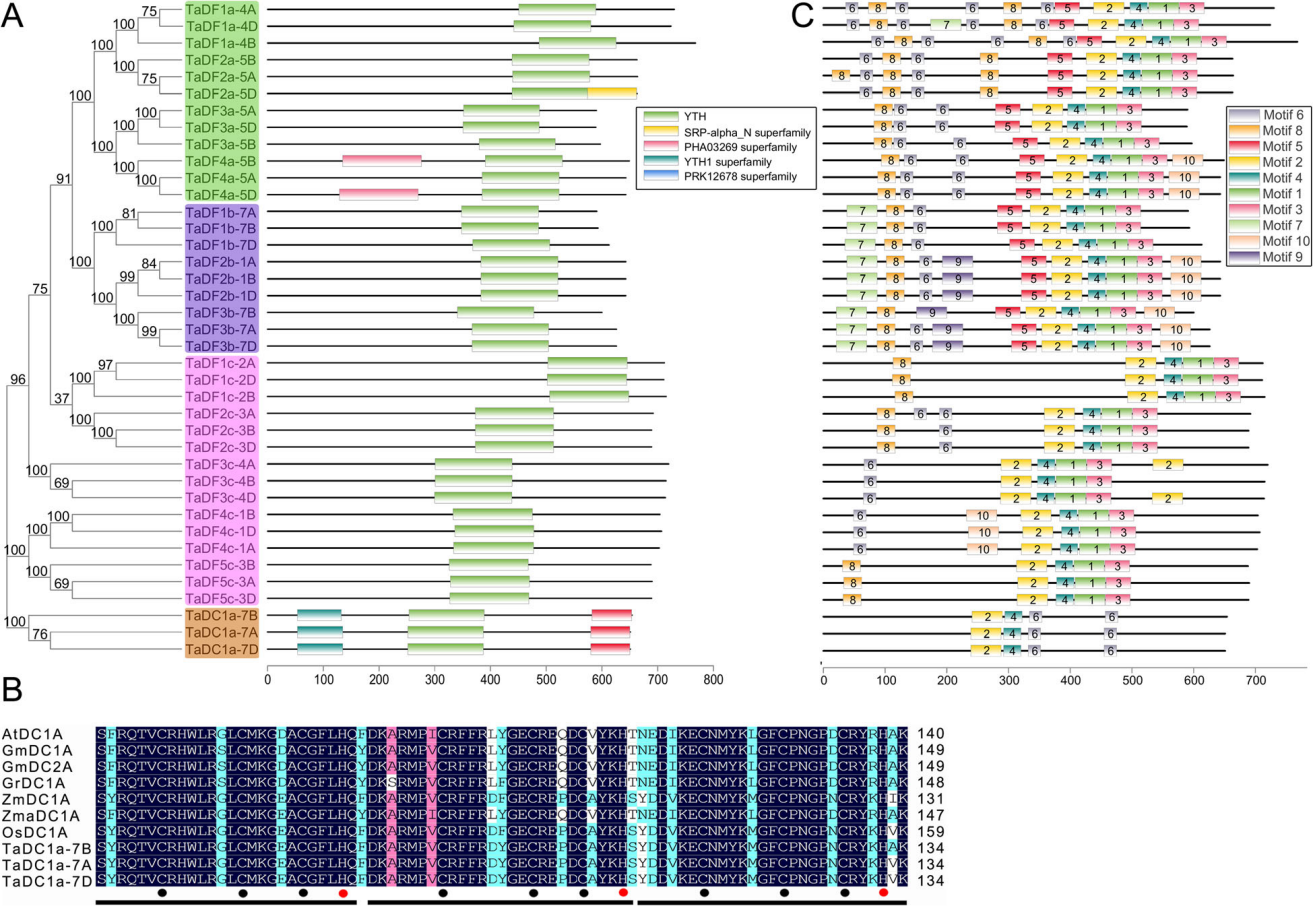
Conserved domains and motif of YTH proteins. **a**. Conserved domains in TaYTHs. **b**. Sequence alignments among the YTH1 superfamily domain regions of the type-a YTHDCs in *A. thaliana, G. max, Gossipium raimondii, Z. mays, Zostera marina, O. sativa*, and Wheat. Three repeated C3H1-type zinc fingers in YTH1 superfamily domain were underlined. Black and red dots indicate the conserved Cys and His, respectively. **c**. Conserved motifs of TaYTHs

**Fig. 5:**
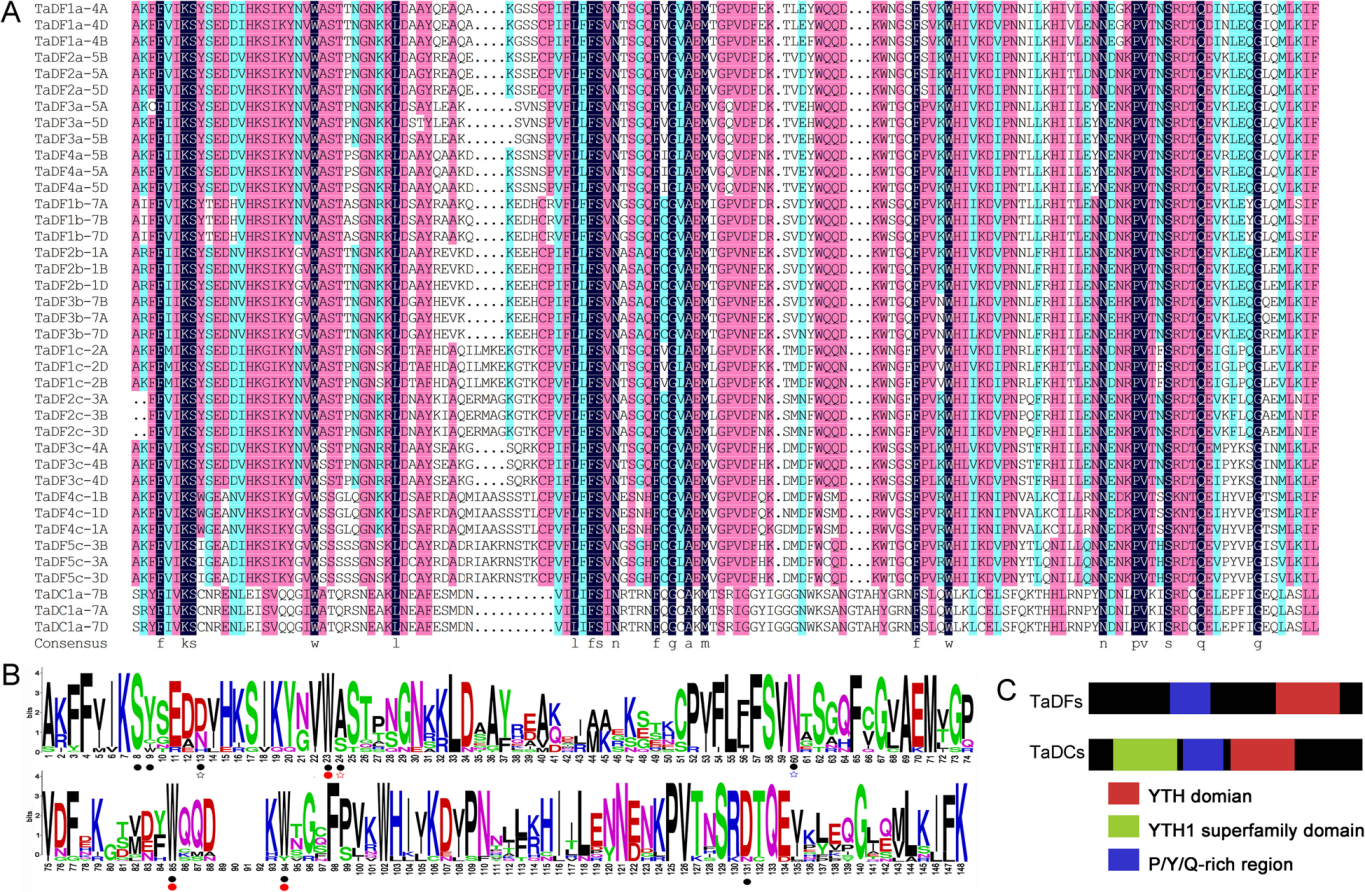
YTH Domains and Y/P/Q-rich region in TaYTHs. **a**. Alignments of the YTH domains of TaYTHs. **b**. Frequency distribution of amino acids in YTH Domains of TaYTHs. The amino acids participating in binding of m6A RNA are indicated with black points below the sequence. The amino acids involved in cage formation are indicated with red points. Amino acids implicated in H bond formation with N1 hydrogen, N6 hydrogen, and 2′-OH group of m6A are indicated with black, red and blue stars, respectively. The numbers on X axis indicate the relative position of amino acids, and the numbers on Y axis represent the conservation of amino acids. The height of a letter indicates its relative frequency at the given position (X-axis) in the motif. **c**. Locations of Y/P/Q-rich regions in TaDFs and TaDCs

Sequence alignments revealed that the TaYTH proteins only share 30.85% identity, and the highly conserved regions are located in the YTH domains with 69.57% identity (Additional file 4), which is similar to the YTH domain-containing proteins of other species [6]. In most of the TaDF proteins, the YTH domain is the only recognizable module at their C-terminus (Fig. 4a) that is similar with those of other species [11]. In contrast, in addition to the YTH domain at their middle parts, all three TaDC proteins harbor two additional domains, an N-terminal YTH1 superfamily domain and a C-terminal PRK12678 superfamily domain. The YTH1 superfamily domain is composed of three repeated C3H1-type zinc fingers [13] (Fig. 4b). All these TaDC proteins were classified as type-a YTHDC (Fig. 1). The type-a YTHDCs in other plants also possess the YTH1 superfamily domain at their N-terminus, but their C-terminus harbors different types of domains (Additional files 5 and 6). In contrast, the type-b YTHDCs in dicotyledonous plants do not contain additional domains besides the YTH domain, which is like that of the YTHDC1s in animals (Additional files 5 and 6). To further analyze the structural diversity in the TaYTH proteins, the conserved motifs in TaYTHs were identified using the online tool MEME (http://meme-suite.org/tools/meme) (Additional file 7). All the TaYTH proteins contained motifs 2, 4, and 6. In the TaDFs, the YTH domain is composed of three motifs (2, 4, and 1) and followed by motif 3 at their C-termini. In contrast, the YTH domain of TaDCs consists of three motifs (2, 4, and 6) and is followed by an additional motif 6 at their C-termini (Fig. 5c). Upstream of the YTH domain, motifs 5, 6, and 8 were shared by subgroup TaDFa; in contrast, they are comprised of motif 5, 6, 7 and 8 in subgroup TaDFb and motif 6 and/or 8 in subgroup TaDFc (Fig. 4c).

An aromatic cage pocket in the YTH domain of YTHDF and YTHDC is utilized to bind the methylysine residue of m6A [6, 8]. This positively-charged pocket is formed by the side chains of tryptophan (W411), tryptophan (W465), and tryptophan (W470) in human YTHDF1; and tryptophan (W377), tryptophan (W428), and leucine (L439) in human YTHDC1. In this study, we found that the aromatic cage in the TaDFs is composed of tryptophan, tryptophan, and tryptophan (WWW), but the cage in TaDCs is composed of tryptophan, tryptophan, and tyrosine (WWY) (Fig. 5a and b). In addition to the methylation-dependent interactions, m6A also forms base-specific hydrogen bonds with the YTH proteins [7]. A comparison of the binding affinity between YTHDF1 and YTHDC1 with m6A revealed that the asparagine (N367) in YTHDC1 forms a hydrogen bond with N^1^ of m6A that is stronger than the corresponding aspartic acid (D401) residue with N^1^ of m6A in YTHDF1. In this study, we found that all the TaDFa, six TaDFb and three TaDFc proteins used aspartic acid to form a hydrogen bond with N^1^ of m6A; however, aspartic acid has been replaced by asparagine in six TaDFb, three TaDFc, and three TaDC proteins (Fig. 5a and b). Additionally, histidine is used in the corresponding position in three TaDFb proteins (Fig. 5a and b), and this mode was not found in other YTH proteins identified in plant species [15]. The N-termini of the TaDF proteins possess low-complexity regions containing Y/P/Q-rich regions (Fig. 5c, Additional file 8); however, the Y/P/Q-rich regions in TaDCs were located between the zinc finger repeat (YTH1 superfamily domain) and the YTH domain (Fig. 5c).

### Expression analysis of the *TaYTH* genes in different tissues/organs and under abiotic stresses

**Fig. 6:**
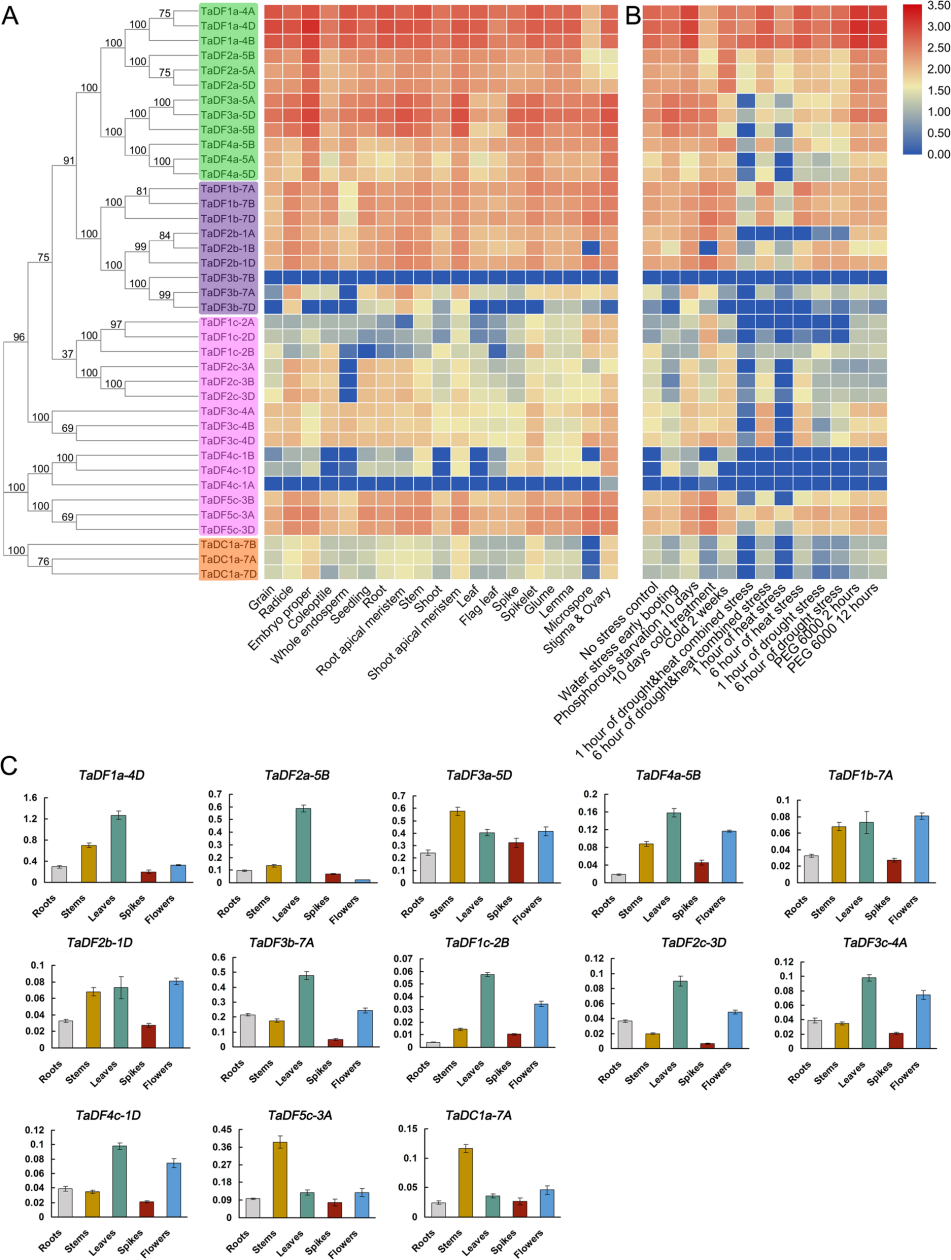
Expression patterns of *TaYTH* genes in different tissues/organs and in response to abiotic stress. **a**. Heat map showing the expression of *TaYTH* genes in different tissues/organs at vegetative growth and reproductive stages. **b**. Heat map showing the expression of *TaYTH* genes in response to abiotic stress. **c**. qRT-PCR analysis of *TaYTH* expression in roots, stems, leaves, spikes and flowers in winter wheat cultivar Jimai 22. The vertical axes indicate the relative expression levels

To analyze the patterns of *TaYTH* expression, we downloaded the RNA-Seq data of wheat from the Wheat Expression Browser website (http://www.wheat-expression.com/) [24]. The expression data of 19 tissues/organs (grain, radicle, embryo proper, coleoptile, endosperm, seedling, root, root apical meristem, stem, shoot, shoot apical meristem, leaf, flag leave, spike, spikelet, glume, lemma, microspore, stigma, and ovary) at different development stages and abiotic/biotic stress conditions were included in our analysis. The results revealed that all the *TaDFa* and *TaDFb* genes were highly expressed in the 19 tissues/organs with the exception of *TaDF3bs* (Fig. 6a). In contrast, the levels of expression of *TaDFcs* and *TaDCas* were lower in various tissues/organs with the exception of *TaDF3cs* and *TaDF5cs* (Fig. 6a)*.* Most of the *TaYTH* genes exhibited obvious changes of expression in response to abiotic stresses, such as phosphorus starvation, cold stress, and heat stress (Fig. 6b). In addition, the expression of *TaDF3b-7B* and *TaDF4c-1A* are low in all the tissues/organs and stress conditions analyzed (Fig. 6a and b). *TaDFa* and *TaDFb* genes showed highly expressed under biotic stresses with the exception of *TaDF3b-7B* (Additional file 9)*.* In contrast, *TaDFc and TaDCa* are low expressed except that *TaDF5c* is high expression under biotic stresses (Additional file 9).

To confirm the tissue-expression of the *TaYTH* genes in common wheat, we performed qRT-PCR analyses in five different tissues/organs of winter wheat cultivar Jimai22, including roots, stems, leaves at vegetative development, and spikes and flowers at reproductive development. A total of 13 genes (*TaDF1a-4D, TaDF2a-5B, TaDF3a-5D, TaDF4a-5B, TaDF1b-7A, TaDF2b-1D, TaDF3b-7A, TaDF1c-2B, TaDF2c-3D, TaDF3c-4A, TaDF4c-1D, TaDF5c-3A,* and *TaDC1a-7A*) and one of each set of homeoalleles were included in our analysis. *TaDF1a-4D* exhibited the highest level of expression in five tissues among the 13 *TaYTH* genes (Fig. 6c), which is consistent with the results based on the RNA-Seq data (Fig. 6a). Our results revealed that 11 out of 13 *TaYTH*s were expressed at relatively higher levels in leaves with the exception of *TaDF5c-3A*, and *TaDC1a-7A* (Fig. 6c). In addition, the levels of expression of *TaDF3a-5D, TaDF1b-7A, TaDF5c-3A* and *TaDC1a-7A* in the stems and *TaDF3a-5D, TaDF4a-5B, TaDF1b-7A, TaDF2c-3D,* and *TaDF3c-4A* in the flowers were at relatively higher levels. In contrast, only *TaDF3b-7A* and *TaDF3c-4A* exhibited a higher level of expression in the roots and spikes, respectively (Fig. 6c).

## Discussion

In our study, a total of 39 putative YTH domain-containing protein family genes were identified in common wheat. Yue et al. identified 41 m6A reader proteins in wheat [25], and 38 of them contained the YTH domain. We identified an additional wheat gene, TaDC1a-7D (TraesCS7D02G450100.1), which encodes a protein containing YTH domain at its middle part, and was designated member of the wheat YTHDC group (Table 1). In the study by Yue et al. (2019) [25], TaCPSF30–2 (TraesCS6A01G138900.1) and TaCPSF30–5 (TraesCS6B01G167200.1) are designated YTHDC proteins. However, no YTH domain was identified in these two proteins. *TaCPSF30–2* and *TaCPSF30–5* were localized in chromosomes 6A and 6B [25]. However, in our results, no *YTH* gene localized in any form of chromosomes 6 (A, B, and D) was identified. These discrepancies might have resulted from the different annotation of version 1 and 1.1 of the wheat genome (IWGSC RefSeq Annotations v1.0 and v1.1). A chromosomal distribution map revealed that the *TaYTH* genes are dispersed across all the chromosomes of all three wheat sub-genomes with the exception of chromosome 6. A similar pattern of dispersion was also found in the *YTH* genes in *A. thaliana O. sativa* and *M. domestica*, but it is different from those in *C. sativus*, in which the *CsYTH* genes are located on three out of seven of the *C. sativus* chromosomes [13].

It has been reported that a conserved mechanism was utilized by the YTH domain proteins YTHDF and YTHDC to recognize m6A, an aromatic cage that recognizes the methyl moiety of m6A [6, 8]. The YTHDF family of proteins has a WWW cage, while the YTHDCs have a WWL-type cage in humans. In this study, we found that the aromatic cage in TaDFs is comprised of WWW, but the cage in TaDCs is comprised of WWY. This rule is employed by the YTHDC proteins from other plant species. These analyses indicated that the plant YTHDCs are different from the animal YTHDCs in m6A binding. In animals, two types of YTHDCs were identified, YTHDC1 and YTHDC2. In YTHDC1, the YTH domain is located in the central part of protein, while the YTH domain is located at the C-terminus of YTHDC2. In plant species, two types of YTHDC were also identified. However, the YTH domains in plant YTHDCs were all localized in the central part of proteins, which is similar to that of the animal YTHDC1s. This suggests that the YTHDCs in plants could share similar molecular mechanisms with those of the animal YTHDC1s. In humans, YTHDC1 mediates pre-mRNA splicing by interacting with splicing factor [26]. Pre-mRNA splicing is involved in the regulation of plant development, sexual reproduction, and abiotic stress responses [27–35]. Thus, the functions of plant YTHDCs in pre-mRNA splicing merit further study. Additionally, the N-termini of plant YTHDFs do not possess a zinc finger domain like those found in the plant YTHDCs. This is consistent with the suggestion that, aside from their conserved YTH domain, YTHDCs are unrelated to YTHDFs based on their amino acid sequences [6]. Animal YTHDF proteins harbor YTH domain at their C-termini and a low-complexity region at their N-termini. In this low-complexity region, several P/Q/N-rich patches were found in animal YTHDF proteins. P/Q/N-rich patches also were identified in TaYTHs in this study. A P/Q/N-rich region is suggested to function in the regulation of the stability of m6A-containing mRNAs by localizing the YTH domain-containing proteins to the RNA decay sites [36–38]. m6A forms base-specific hydrogen bonds with YTH proteins that enhance these interactions [7]. In human YTHDF1 and YTHDC1, asparagine or aspartic acid is used to form the hydrogen bond with N^1^ of m6A, although asparagine interacts more strongly with N^1^ of m6A than does aspartic acid. However, in some wheat YTH proteins, histidine might be utilized for the interaction with N^1^ of m6A. Asparagine and aspartic acid are neutral amino acids, while histidine is basic. Thus, the binding of m6A with YTH proteins in wheat might have substantial differences with those in other plant and animal species [6, 15]. Human YTHDC2 is a nucleocytoplasmic protein that contains a DEAD-box RNA helicase domain in addition to a C-terminal YTH domain. The DEAD-box RNA helicase domain-containing proteins function in unwinding double stranded RNA [39]. In fact, the YTHDC2 proteins play roles in unwinding 5′-UTR and increasing the translational efficiency of the target gene [40]. However, no DEAD-box RNA helicase domain-containing YTH proteins have been identified in the plant kingdom. Thus, whether YTH-involved unwinding is required for m6A recognition by the readers remains unknown.

Wheat is an important crop around the world, which provides more than 20% of the protein and caloric intake of humans. The yield of wheat is closely related with the development and architecture of spikes. Many signaling pathways have been identified that are involved in the regulation of spike development and architecture in wheat [41], during which the regulation of gene expression and hormone signaling play critical roles. In our study, a set of *YTH* genes were expressed at higher levels during the development of spikes and flowers of wheat. Thus, these highly expressed *YTH* genes in the wheat spikes might function in the development and architecture of spikes. The selection and creation of wheat germplasm with tolerance to multiple abiotic and biotic stresses is a key challenge for wheat breeding [42–56]. Our results and those of previous studies reveal that the expression of *YTH* gene is involved in the responses to various stresses and in plant development [10, 13, 15, 17, 21]. Therefore, the function of wheat *YTH* genes in the response to abiotic stresses merits further study, and these *YTH* genes might be valuable for wheat genetic improvement.

## Conclusions

In this study, 39 *TaYTH* genes were identified in common wheat (*Triticum aestivum* L.). A phylogenetic analysis revealed that the TaYTHs could be divided into two groups, YTHDF (TaDF) and YTHDC (TaDC). The chromosomal location and exon-intron structure of the genes, and the motif distribution and domain organization of the proteins were analyzed. In addition, the aromatic cage pockets of TaYTH proteins were predicted. The aromatic cage pocket of TaDFs is composed of tryptophan, tryptophan, and tryptophan (WWW). In contrast, this pocket is composed of tryptophan, tryptophan, and tyrosine (WWY) in TaDC proteins. In addition to the general aspartic acid or asparagine residues used to form a hydrogen bond with N^1^ of m6A, some TaDFb proteins might utilize histidine in this role. The results of the analysis of expression revealed that the *TaDFa* and *TaDFb* genes were highly expressed in various tissues/organs. In addition, the expression of *TaYTH* genes is changed in response to various abiotic stresses. Our results provide the foundation for the functional analysis of *TaYTH*s in the future.

## Methods

### Identification of the *YTH* genes in wheat

The YTH protein family in wheat was identified as described by Yan et al. [57]. The conserved sequence of the YTH domain was used to make a Hidden Markov model and used in BLAST homology searches. The reference genome sequence and annotations (GFF files) of wheat were downloaded from the wheat genome Database (IWGSC RefSeq v2.0) at Ensemble Plants (http://plants.ensembl.org/Triticum_aestivum/). The conserved YTH domain was also used to BLASTp the YTH proteins encoded by the wheat genome on the website http://plants.ensembl.org/Multi/Tools/Blast [8]. Additionally, BLASTP analysis against the local wheat protein database (E-value < 1 × 10^− 5^) was performed by using the *Arabidopsis* and rice YTH domain-containing protein sequences as queries (Additional file 2).

### Phylogenetic analysis, gene structure, and chromosomal locations

ClustalX2.1 was used for multiple sequence alignments with default parameters. A maximum likelihood phylogenetic tree was constructed using MEGA 6.0 (https://www.megasoftware.net/) with 1000 bootstrap replicates. The phylogenetic tree was visualized by FigTree (http://tree.bio.ed.ac.uk/software/). TBtools software [58] and the online analytical tool KnetMiner (https://knetminer.rothamsted.ac.uk/Triticum_aestivum/) were used to map the locations of HTY genes in chromosomes and construct the map of exon-intron structure.

### Amino acid sequence analysis

Online tool PSORT (https://www.psort.org/) was used to predict the subcellular localization of the TaYTH proteins. The molecular masses and isoelectric points of the TaYTH proteins were predicted using the web tool ExPASy (https://web.expasy.org/compute_pi/). The domains of TaYTH proteins were analyzed using the web tools CDD (https://www.ncbi.nlm.nih.gov/) and ExPASy (https://prosite.expasy.org/). Finally, the domain graphs were visualized using TBtools [48]. The motif compositions of TaYTHs were identified by MEME (http://meme-suite.org/tools/meme) and exhibited using TBtools software. Multiple protein sequence alignments were performed using DNAMAN8. Sequence logos of the conserved motifs of proteins were created using Weblogo (http://weblogo.berkeley.edu/logo.cgi).

### Digital gene expression pattern analysis

The transcript per million values of expression of *TaYTH* genes in 19 tissues/organs (grain, radicle, embryo proper, coleoptile, endosperm, seedling, root, root apical meristem, stem, shoot, shoot apical meristem, leaf, flag leave, spike, spikelet, glume, lemma, microspore, stigma and ovary) and in response to various stresses were downloaded from the Wheat Expression Browser (www.wheat-expression.com) [24]. Heat maps that showed the relative expression levels were constructed using TBtools.

### Plant materials and growth conditions

The common wheat used in this study is Jimai22, a winter wheat cultivar that is widely planted in North China. The seeds of Jimai22 were provide by Dr. Yu Xiu Dong (Shandong Agricultural University, China). The wheat seedlings were grown in a greenhouse at 22 °C with a 16 h light/8 h dark cycle at Shandong Agricultural University. The roots, stems, and leaves were sampled from 7-day seedlings. To sample the spikes and flowers, Jimai22 seeds were first vernalized in soil for 30 days at 4 °C. The vernalized seedlings were then transferred into greenhouses. The spikes and flowers were sampled from the plants grown for 30 days and 40 days in a greenhouse, respectively.

### Expression of the *TaYTH* genes analyzed by qRT-PCR

Total RNA was extracted using the TRIzol reagent (Invitrogen, Beijing, China) as described by Zhao et al. [59]. The total RNA was reverse transcribed using a PrimeScript RT Reagent Kit (TakaRa, Dalian, China) according to the manufacturer’s instructions. The first strand cDNAs for qRT-PCR were synthesized using Synthesis SuperMix (TransGen, Beijing, China). The qRT-PCR amplifications were performed as described by Zhao et al. [60]. Three biological replicates and three technical replicates were utilized. The Actin gene was used as an internal control. The sequences of the primers used in this study are listed in Additional file 10.

## Supplementary information

**Additional file 1.** Sequences of TaYTHs.

**Additional file 2.** Sequences of YTHs used for the construction of phylogenetic tree.

**Additional file 3 **Gene pairs between *M. truncatula* and wheat, barley and wheat, rice and wheat, maize and wheat, *B. distachyon* and wheat.

**Additional file 4.** Sequence alignments among the identified TaYTH proteins.

**Additional file 5.** Organization and distribution of the conserved domain in YTHDC1s in animals and YTHDCs in plants.

**Additional file 6.** Sequences of animal YTHDC1s and plant YTHDCs.

**Additional file 7.** Conserved motifs of TaYTHs.

**Additional file 8.** Y/P/Q-rich region in TaYTHs.

**Additional file 9 **Heat map showing the expression of *TaYTH* genes in response to biotic stress.

**Additional file 10.** Primers used in this study.

## Data Availability

All data generated or analyzed in this study are included in this published article and its Additional files. The datasets generated and analyzed during the current study are available from the corresponding author on reasonable request.

## Abbreviations

MW: Molecular weight
m6A: N6-Methyladenosine
pI: Isoelectric point
YTH: YT521-B homology
