# Supplementary material for: Genome-wide identification and expression analysis of YTH domain-containing RNA-binding protein family in common wheat
Source: BMC Plant Biol. 2020 Jun 23;20:351. doi: 10.1186/s12870-020-02505-1 (PMC7384225; doi:10.1186/s12870-020-02505-1)
Supplement: Supplementary file 4 — Additional file 4. Sequence alignments among the identified TaYTH proteins. [file 12870_2020_2505_MOESM4_ESM.docx]

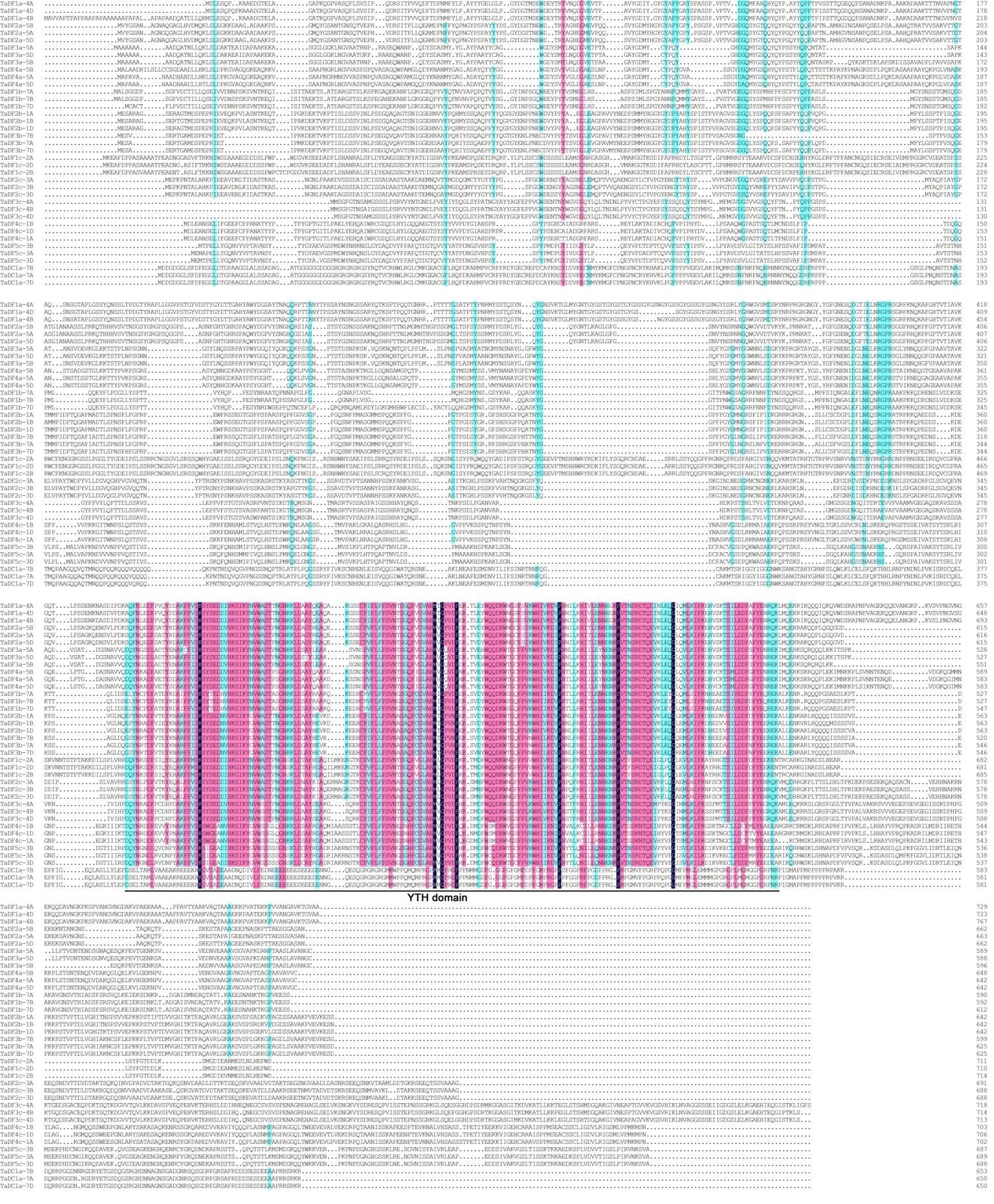


Additional file 4. Sequence alignments among the identified TaYTH proteins. The conserved YTH domains were underlined.
