# Supplementary material for: Genome-wide identification and expression analysis of YTH domain-containing RNA-binding protein family in common wheat
Source: BMC Plant Biol. 2020 Jun 23;20:351. doi: 10.1186/s12870-020-02505-1 (PMC7384225; doi:10.1186/s12870-020-02505-1)
Supplement: Supplementary file 5 — Additional file 5. Organization and distribution of the conserved domain in YTHDC1s in animals and YTHDCs in plants. [file 12870_2020_2505_MOESM5_ESM.docx]

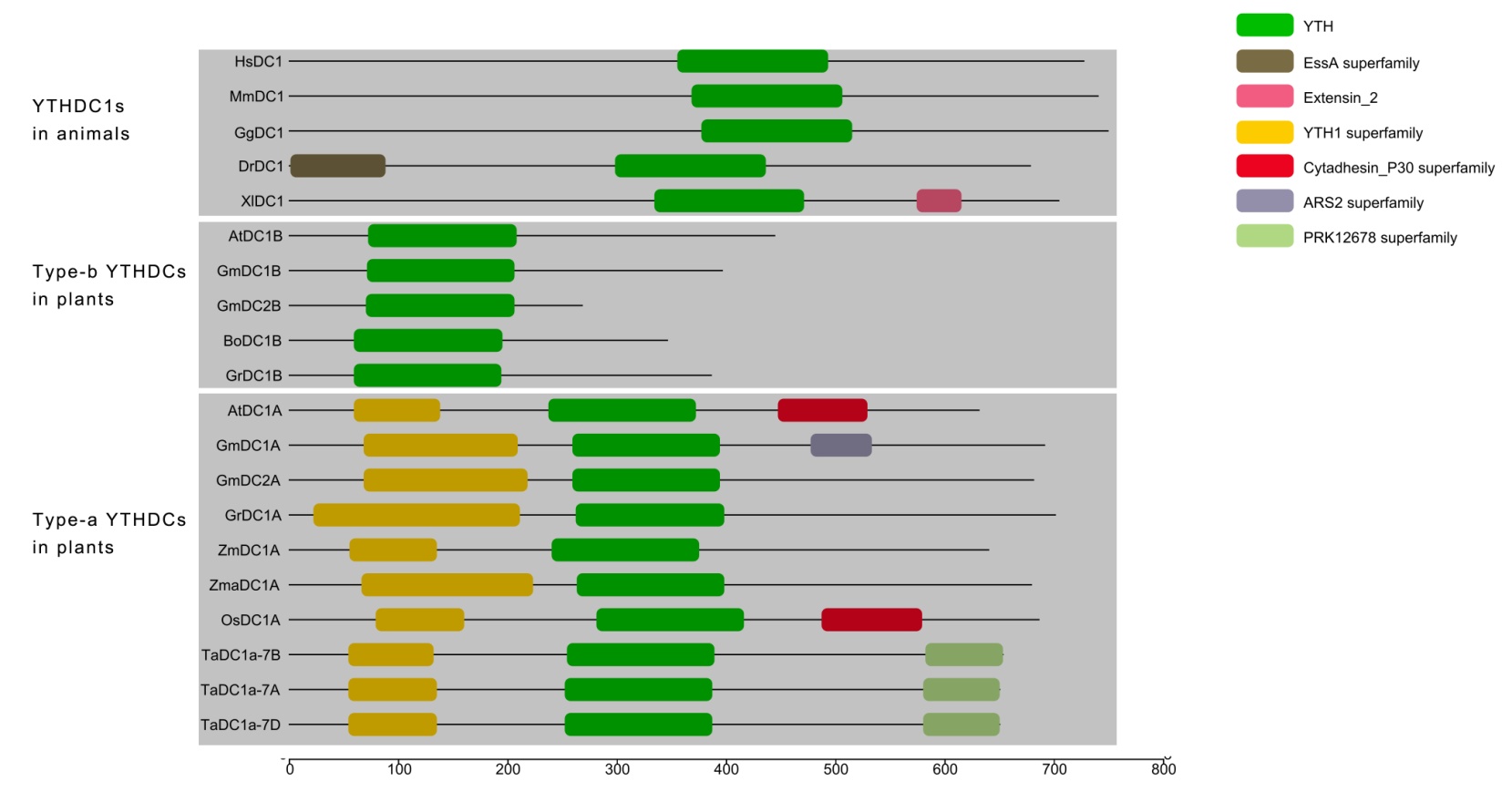


Additional file 5 Organization and distribution of the conserved domain in YTHDC1s in animals and YTHDCs in plants. Domains were predicated using the webtool CDD/SPARCLE (<http://www.ncbi.nlm.nih.gov/Structure/cdd/wrpsb.cgi>) based on the sequences (Additional file 4).
