# Supplementary figures and images for: Genome-wide identification and expression analysis of YTH domain-containing RNA-binding protein family in common wheat
Source: BMC Plant Biol. 2020 Jun 23;20:351. doi: 10.1186/s12870-020-02505-1 (PMC7384225; doi:10.1186/s12870-020-02505-1)

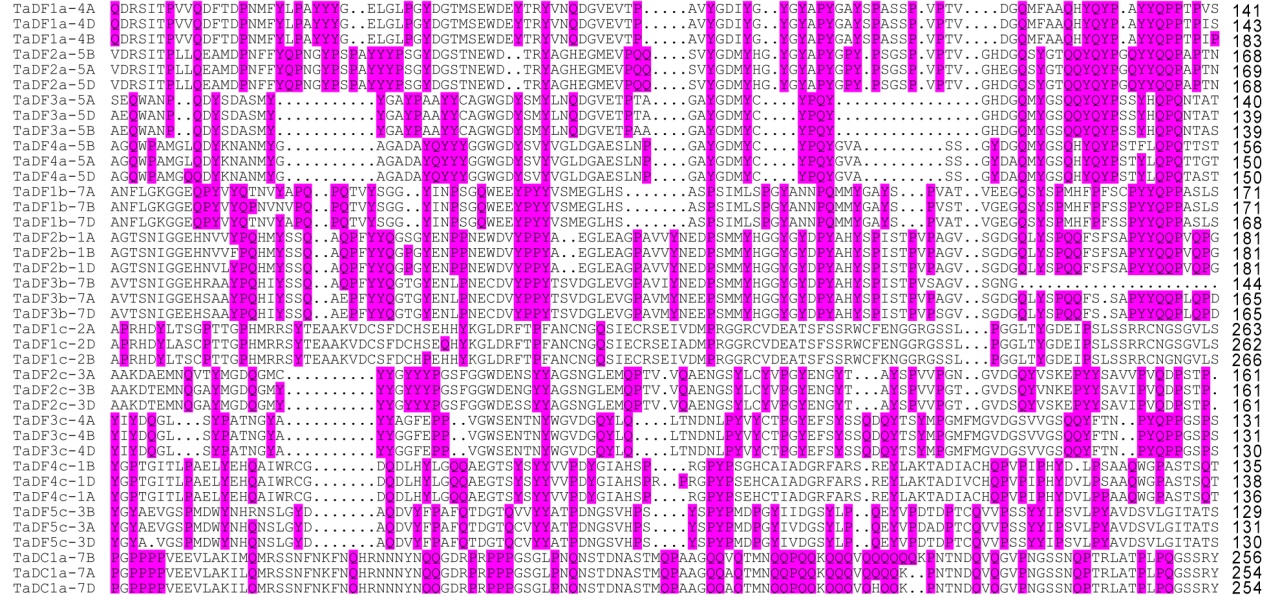


Additional file 8. Y/P/Q-rich region in TaYTHs.

The Y/P/Q amino acids are highlighted in red.

Supplement: Supplementary file 8 — Additional file 8. Y/P/Q-rich region in TaYTHs. [file 12870_2020_2505_MOESM8_ESM.docx]

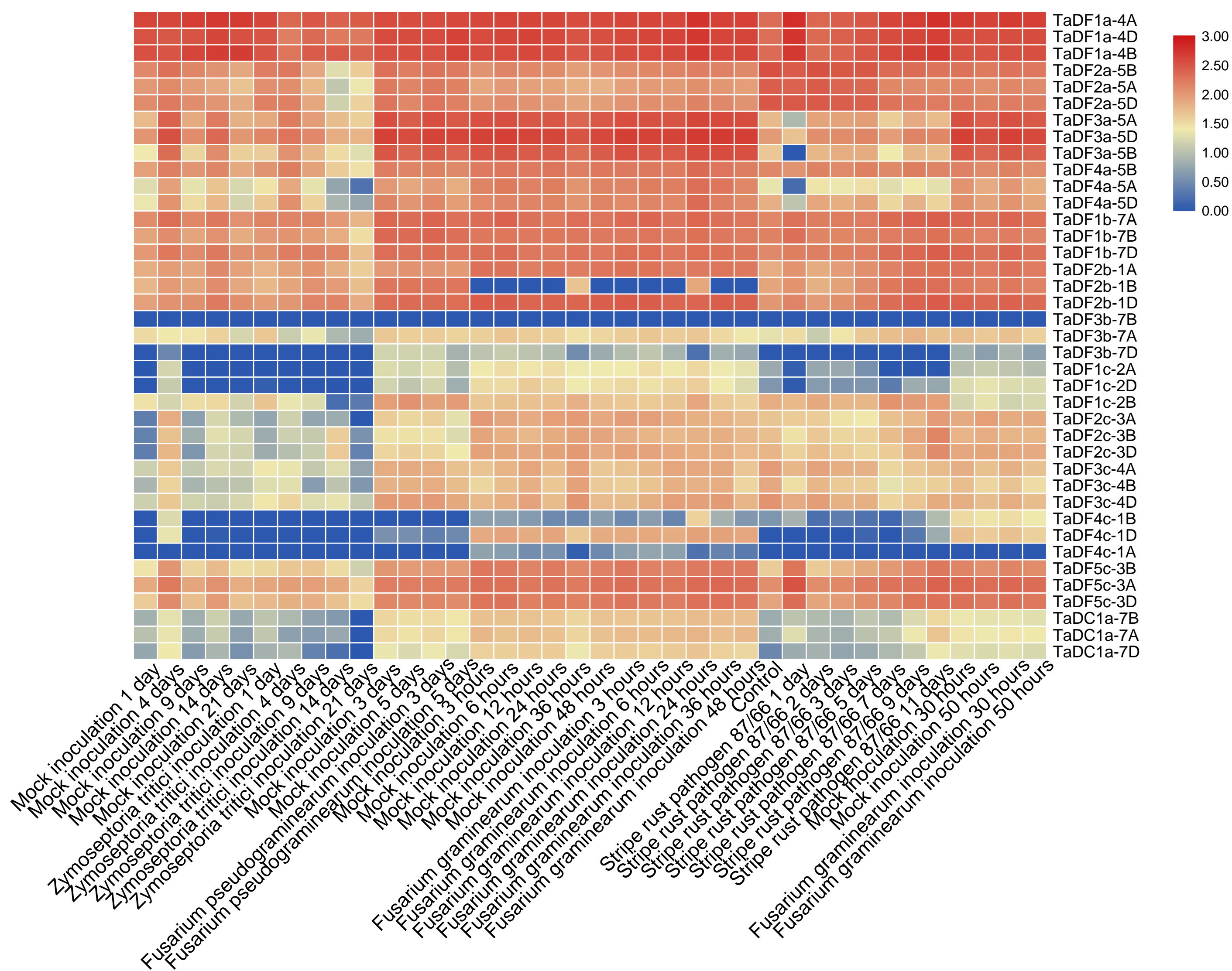

Supplement: Supplementary file 9 — Additional file 9 Heat map showing the expression of TaYTH genes in response to biotic stress. [file 12870_2020_2505_MOESM9_ESM.pdf]
